# Contact-free Measurement of Heart Rate Variability via a Microwave Sensor

**DOI:** 10.3390/s91209572

**Published:** 2009-11-30

**Authors:** Guohua Lu, Fang Yang, Yue Tian, Xijing Jing, Jianqi Wang

**Affiliations:** 1 Department of Biomedical Engineering, Fourth Military Medical University, Xian, 710032, China; E-Mails: lugh1976@fmmu.edu.cn (G.L.); fmmujxj@163.com (X.J.); 2 Department of Experimental Teaching Centre, Fourth Military Medical University, Xian, 710032, China; E-Mail: yfjiaoxue@yahoo.com.cn; 3 Department of Medical Instruments, Xijing Hospitals, Xian, 710032, China; E-Mail: touchty@fmmu.edu.cn

**Keywords:** heart rate variability, contact-free, heartbeat, microwave sensor

## Abstract

Measures of heart rate variability (HRV) are widely used to assess autonomic nervous system (ANS) function. HRV can be recorded via electrocardiography (ECG), which is both non-invasive and widely available. However, ECG needs three electrodes touching the body of the subjects, which makes them feel nervous and uncomfortable, thus potentially affecting the recording. Contact-free detection of the heartbeat via a microwave sensor constitutes another means of determining the timing of cardiac cycles by continuous monitoring of mechanical contraction of the heart. This technique can measure the heartbeat without any electrodes touching human body and penetrate the clothes at some distances, which in some instances may prove a practical basis for HRV analysis. Comparison of 5-minute recordings demonstrated that there were no significant differences in the temporal, frequency domains and in non-linear dynamic analysis of HRV measures derived from heartbeat and ECG, which suggested this technique may prove a practical alternative to ECG for HRV analysis.

## 1. Introduction

Analysis of Heart Rate Variability (HRV) is a powerful tool used to evaluate the regulation of cardiac activity by the autonomic nervous system (ANS). Since its inception HRV has been proven to index fetal distress[1], reveal diabetic neuropathy [2] and uncover ANS pathology [3]. Importantly, HRV has also been shown to predict the mode of death in chronic heart failure[4], raising the prospect that HRV may prove a valuable guide to clinical intervention in cardiovascular disease [5].

HRV measurements are established by analysis of the temporal relationship between successive heartbeats. Traditionally this signal is determined by electrocardiography (ECG) recordings using three Ag/AgCl electrodes attached to specific anatomical positions, in accordance with Einthoven [6]. During the recording, on the one hand the subjects may feel uncomfortable and limited, and on the other hand, common sources of noise are those generated by physiological processes, including electromyography contamination, signal interference and respiration induced baseline drift, as well as those generated by non-physiological influences such as power line interference and electrode contact movement.

Contact-free detection of life signatures via a microwave sensor is a new technique, which can penetrate some non-metal media like wood, clothes and remotely sense the respiration and heartbeat signals without any electrodes or sensors touching the body of human subjects [7-12]. Early applications of this technique were specially used in searching for survivors after earthquakes or snow avalanches due to the good penetration capability of microwaves [13], and some of the published papers [14,15] and patents [16,17] have focused on the detection of movement due to breathing or heartbeat in order to estimate their rate.

The work described in the paper was aimed at evaluating the use of this contact-free microwave sensor for quantitative measures of heart rate variability, as a more powerful tool for the regulation of cardiac activity than heart rate or respiratory rate, using the signal analysis techniques applied to 3-lead ECG signals in healthy subjects. Here we show under controlled research conditions that measures of ANS function derived from the ECG system and the microwave sensor are similar by comparing 5-minute heartbeat and ECG recordings to compute HRV in time, frequency domains and using non-linear dynamic indices.

## 2. Description of the Microwave Sensor

**Figure 1.**
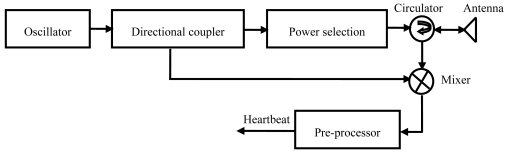
The block scheme of the microwave sensor.

The block scheme of the custom-developed contact-free microwave sensor [18] is shown in Figure 1. The electromagnetic wave was generated by the oscillator via a directional coupler. The oscillator, made of a GaAs Gunn diode was chosen to meet the demands of low noise and low cost, can also provide linear continuous waves. The oscillator operated at 35 GHz and the maximum transmission power was about 10 mW. The microwave beams were radiated through a two-way parabolic antenna via a circulator. The gain of the antenna is 17 dB, and the beam width is 9 degree in both the horizontal and vertical directions. Another signal from the directional coupler acted as a local oscillatory signal for the receiver. The echo signal was received by the antenna and then passed through the circulator to get into the mixer where it was mixed with the local oscillatory signal. The output of the mixer was conditioned by a pre-processor, composed of an amplifier with the gain of 60 dB, an analog low-pass filter with cutoff frequency of 0.5 Hz and slope of 12 dB/octave, an analog low- pass filter with cutoff frequency of 3.3 Hz and slope of 12 dB/octave, and a 50 Hz notch filter. The custom developed rechargeable power supply could provide 5 Volt up to 5 Amp Hours and the power consumption of the microwave sensor was less than 3 Watts. Thus the sensor could continuously work over 8 hours after the lithium batteries were fully recharged. The output of the pre-processor was called the heartbeat signal.

## 3. Signal Recording and Analyzing

For recording of the electrocardiogram, disposable Ag/AgCl resting ECG electrodes (Red Dot™-2352; 3M Company; MN, USA) were attached to the lower of left chest (‘Ground’), upper of right chest (‘Negative’) and the upper of left chest (‘Positive’). Wires from the electrodes (LEAD108A, ECG100C; BIOPAC Systems Inc.; Goleta, CA, USA) were attached to an ECG monitoring system (ECG100C; BIOPAC Systems Inc.).

Outputs of the ECG system and the microwave sensor were connected to a 16-channel A/D converter (MP150; BIOPAC Systems Inc.), which was in turn directly connected to a desktop computer through a 10M / 100Mbps Ethernet adapter. All the signals were sampled at a frequency of 1000 Hz simultaneously recorded for 7 minutes using the AcqKnowledge software package (v 3.8.1; BIOPAC Systems Inc.) and saved to a text file for further processing.

Sixteen volunteers (16 males; 23.0 ± 4.0 years (mean ± S.D.)) participated in this study. Ethic Committee of the Fourth Military Medical University approved the study. All subjects were healthy and informed consent was obtained prior to their participation. Subjects sat on a chair and remained still throughout the recording period when they were instructed to minimize their movement. The distance between the subject and the antenna, the ECG monitoring system was 3.6 m.

### 3.1. Extraction of HRV Signals Derived from ECG and Heartbeat

An experienced researcher (GL) selected 5-minute ECG and heartbeat segments with minimal artefact. HRV analyses were performed with purpose-written algorithms, using the MATLAB software package (MATLAB version 6.5; The MathWorks, Inc; Natic, MA, USA).

For the ECG recordings, the extraction method incorporated a peak detection algorithm that found the time of occurrence of each QRS complex contained a Q wave, an R wave, and an S wave in the filtered ECG signal [19], and then the durations between successive peak locations were calculated to produce a time series of R-R intervals (RRIs).

For the heartbeat recordings, a neighboring searching method was used to derive the H valley events from the amplitude of heartbeat signals and then the successive detected H valleys intervals (HHIs) were calculated. All of the RRI and HHI time series underwent an initial automated editing process before a careful manual editing was performed by visual inspection.

### 3.2. Measuring Parameters in RRI and HHI Recordings

#### 3.2.1. Time Domain Parameters

Four parameters were calculated from time domain RRI and HHI recordings [20] the mean interpulse interval (mean NN), the standard deviation of the interpulse intervals (SDNN), the square root of the mean squared differences of successive interpulse intervals (RMSSD) and the proportion of differences of successive interpulse interval exceeding 50 ms, known as pNN50; this was derived by the number of interpulse interval exceeding 50 ms dividing by the total number of interpulse intervals.

#### 3.2.2. Frequency Fomain Parameters

The RRI and HHI sequences were cubic interpolated and evenly re-sampled at 4 Hz. Then low frequency (LF) power (0.04–0.15 Hz), high frequency (HF) power (0.15–0.4 Hz) and the ratio of LF to HF power were calculated in accordance with previously published standards for the spectral analysis of HRV[20], yielding three frequency domain measures. Power frequency (Hz) was converted to ms^2^ using the Fast Fourier Transform (FFT) employing 1,024 points using software developed in-house.

#### 3.2.3. Poincaré Parameters

The Poincaré plot is one of the most widely used techniques for nonlinear HRV analysis, it is a plot of each RR interval against the previous one. From a Poincaré plot, two non-linear parameters SD1 and SD2 can be calculated [21]:

(1) $$\text{SD}1=\sqrt{\frac{1}{2}\text{Var}(x_{n+1}-x_{x})}$$

(2) $$\text{SD}2=\sqrt{2{\text{SDNN}}^{2}-\frac{1}{2}SD_{1}^{2}}$$

where *x* represents the HHI or RRI sequences, symbol *Var* is the variance of the differences in successive RRI or HHI, SDNN is the standard deviation of the RRIs or HHIs. SD1 represents short term beat-to-beat variability of the data and SD2 is long term beat-to-beat variability.

### 3.3. Statistical Analysis

To assess how similar HRV parameters derived from heartbeat compared with those derived from the ECG, one-way ANOVA was performed. Before analyses, raw values of all variables were examined for deviations from normality by the Kolmogorov–Smirnov test. Statistical analysis was performed in SPSS^@^ (v13, SPSS Inc.; Chicago, IL, USA) and graphs were plotted using Origin^@^ (v7.5776, Northampton, MA, USA).The level of significance was set at p < 0.05 (two-tailed).

## 4. Experimental Results

**Figure 2.**
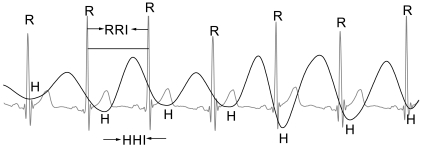
Waveforms of ECG and heartbeat.

Waveforms of 5 second segments of ECG and heartbeat signals from a representative subject are shown in Figure 2. The line in grey is the ECG signal and RRI is the interval of the successive R waves. The line in black displays the heartbeat signal and HHI is the interval of the successive H waves. Because there exits time delay in contact-free heartbeat detection, the R wave and H wave can not be aligned.

**Figure 3.**
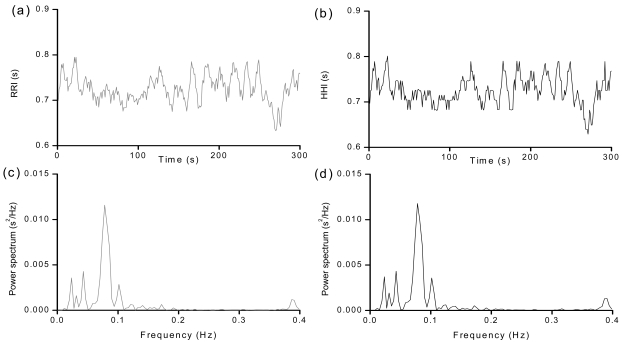
RRIs and HHIs with their power spectrum analysis. Left column is the RRIs derived from the ECG recordings; right column is the HHIs derived from heartbeat recordings. The top panels indicate the RRIs and HHIs ((a)-(b)). The lower two panels show the power spectrum of RRIs and HHIs ((c)-(d)).

The RRIs and HHIs with their power spectrum analysis from 5 minute ECG recording and heartbeat recording of the subject above are displayed in Figure 3. The left column in grey displays the RRI waveforms and its power spectra analyis [(a) and (c)]. The R-R interval is 0.73 ± 0.02 s (mean ± S.D.), and the mean heart rate is 83 beat/min. The peak frequency in the-low frequency (LF; 0.04–0.15 Hz) and the high-frequency (HF) component is 0.08 Hz and 0.39 Hz as shown in Figure 3(c).The power of the LF and HF is 228.60 ms^2^ and 30.40 ms^2^, and the ratio of the logarithm of LF power to the logarithm of HF power is 1.59. The right column in black shows the HHI waveforms and its power spectra analysis [(b) and (d)]. The H-H interval is 0.73 ± 0.02 s (mean ± S.D.) and the mean heart rate is 83 beat/min. The peak frequency in the-low frequency (LF; 0.04–0.15 Hz) and the high-frequency (HF; 0.15–0.40 Hz) is 0.08 Hz and 0.39 Hz as shown in Figure 3(d).The power of the LF and HF is 238.82 ms^2^ and 36.41 ms^2^, and the ratio of the logarithm of LF power to the logarithm of HF power is 1.53. From the results, the waveforms, the power spectra analysis and measures of HRV derived from the RRIs and HHIs are very similar.

The comparison of the parameters in time domain, Frequency domain and Poincaré plot derived from ECG and heartbeat of the sixteen subjects was showed in Table 1. All of the P value is larger than the significance level, which means there is no significant difference in all of the parameters derived from the ECG recording system and the microwave sensor.

## 5. Discussion

Recent reports suggest that HRV measurement may prove an effective means of detecting early cardiovascular disease [22], as well as for predicting the mode of death in chronic heart failure [4]. Consequently, HRV is attracting increasing interest from clinicians as a potentially valuable indicator for choosing prophylactic cardiovascular interventions.

Traditionally, a 3-lead ECG recording system is used to analyze the HRV measures in time domain, frequency domain and non-linear domain. During the recording of the ECG, noise generated by electromyography contamination, respiration induced baseline drift, as well as the non-physiological influences such as power line interference and electrode contact movement will affect the detection of the QRS complex. Complicated signal processing methods such as wavelet, ICA and adaptive filtering will be used to remove the noised caused by the factors above [23,24].

Thus, a number of researchers have suggested that contact-free life signatures detection via a microwave sensor may be a promising and ubiquitous technique for ambulatory recording of heartbeat and respiration [25]. This paper suggested extraction of the measures of HRV by a 24 GHz microwave system from the back of the subject at a distance of 34 mm, and the results showed the HRV measures from the microwave system strong correlated with that from the ECG system in 2-minute recordings in rest and during doing the simple arithmetic task [26]. As well, our results demonstrated that HRV analysis of signals derived from 5-minute ECG and heartbeat recordings via the microwave sensor are almost identical in healthy subjects.

In our work, a microwave sensor operating at 35 GHz was chosen to extract the HRV measures from the subject at a distance of 3.6 m for possessing several advantages over previously reported systems operating at other microwave frequencies [26,27]. The first reason is that the amplitude of the chest motion due to respiration is expected to be on the order of 10 mm, and due to heart activity on the order of 0.1 mm [28]. Because the accuracy in detecting the heartbeat is directly proportional to the operational frequency [10], the frequency gets higher and the sensitivity to the heartbeat is better. Another important reason is that at 35 GHz, the transmitted signal has no interference with other applications. Moreover, this band is free for use in medical applications whiles the lower frequency band occupied by such as WLAN, Bluetooth, cordless phones and so on. Finally, the shorter the wavelength, the smaller the antennas needed to be integrated.

During the recording of heartbeat via the microwave sensor described in the paper, there are no wires or electrodes directly connected with human body, which there are three benefits comparing with ECG recording system. First, the subject will feel comfortable and relaxed without any physical and psychological burden, which the waveforms of the heartbeat will more objective. Second, the heartbeat can be detected at a distance of several meters by penetrating the clothing, thus the microwave sensor can be attached to the ceiling of a room or somewhere can not be seen by the subject. The procedure of the setup will be easier than the 3-lead ECG recording system, which needs the professional person to operate it. Finally, the microwave sensor combined with the infrared thermometer and the laser Doppler vibrometer may simultaneously monitor the heartbeat, respiration, body temperature and the blood pressure, which can be as the body sensor networks applied in unobtrusive healthcare systems.

However, two things limit the current use in ambulatory recording via currently available microwave sensor. One thing is that the microwave sensor is susceptible to significant motion artifacts that are difficult to eliminate. The advanced signal analysis techniques could be used to reduce motion artifacts overlapped with the heartbeat signal [29,30], which might enable the microwave sensor to be effectively used for long-term recording in mobile patients. Another thing is that the propagation loss of the microwave sensor in free space should be taken into consideration during the recording. According to the theory of microwave [31], the loss is proportional to the square of the distance between the antenna and the subject. In the experiments, the maximum power of the microwave sensor is 10 mW, the distance between the antenna and subject should be lower than 4 m. When the distance is increased, the transmitted power should be increased as well. Otherwise the heartbeat signal can not be detected. With the development of the integrated circuits, high gain and narrow side-lobe antenna in tiny size[32], the microwave sensors can be made very small. Furthermore, the body sensor networks containing the microwave sensor and other contact-free sensors on the technique of MAC/PHY may be widely used in health care monitoring in hospital and family in future.

## 6. Safety

The electromagnetic radiation from the microwave sensor poses no safety threat. The power density level for human exposure can be computed according to the following formula:

(3) $$s(mW/{\text{cm}}^{2})=\frac{\bar{p}.G}{40\pi .r^{2}}$$

where *p̄* (mW)is the average radiating power, G(*dB*) is the gain of the antenna, r (c*m*) is the distance between the antenna and the human subject.

In this study, the maximum radiating power is 10mW and the gain of the antenna is 17 dB. The minimum distance between the human subject and the antenna is 10 cm, so the maximum S is 0.014 mW/cm^2^, which is much lower than the accepted safe power density level of 10 mW/cm^2^ for human exposure at frequencies from 10 to 300 GHz [33].

## Figures and Tables

**Table 1.** The comparison of the parameters in time domain, Frequency domain and Poincaré plot derived from ECG and Heartbeat for group of subjects (n = 16) using one-way ANOVA.

**Parameters**	**ECG**	**Heartbeat**	**P value**

**Time domain**			

Mean NN (ms)	858.00 ± 127.14	857.99 ± 127.20	0.99
SDNN (ms)	50.72 ± 28.67	52.11 ± 28.42	0.39
RMSSD (ms)	47.88 ± 34.59	50.77 ± 34.02	0.25
PNN50 (%)	20.35 ± 17.88	23.00 ± 18.26	0.37

**Frequency domain**			

Log of LF Power	2.43 ± 0.46	2.45 ± 0.45	0.86
Log of HF Power	2.30 ± 0.46	2.34 ± 0.45	0.67
Lg(LF)/Lg(HF)	1.07 ± 0.19	1.06 ± 0.17	0.72

**Poincare' plot**			

SD1 (ms)	34.35 ± 24.70	36.45 ± 24.25	0.27

SD2 (ms)	72.10 ± 33.17	73.06 ± 33.18	0.83

All the values are mean ± SD, p < 0.05
